# Use of *Pleurotus ostreatus* to Enhance the Oxidative Stability of Pork Patties during Storage and In Vitro Gastrointestinal Digestion

**DOI:** 10.3390/foods11244075

**Published:** 2022-12-16

**Authors:** Brisa del Mar Torres-Martínez, Rey David Vargas-Sánchez, Gastón Ramón Torrescano-Urrutia, Marisela González-Ávila, Javier Germán Rodríguez-Carpena, Nelson Huerta-Leidenz, José Angel Pérez-Alvarez, Juana Fernández-López, Armida Sánchez-Escalante

**Affiliations:** 1 Coordinación de Tecnología de Alimentos de Origen Animal (CTAOA), Centro de Investigación en Alimentación y Desarrollo, A.C. (CIAD), Carretera Gustavo Enrique Astiazarán Rosas 46, Hermosillo 83304, Mexico; 2 Consejo Nacional de Ciencia y Tecnología (CONACYT), Av. Insurgentes Sur 1582, México City 03940, Mexico; 3 Centro de Investigación y Asistencia en Tecnología y Diseño del Estado de Jalisco, Av. Normalistas 800, Colinas de La Normal, Guadalajara 44270, Mexico; 4 Unidad Académica de Medicina Veterinaria y Zootecnia, Universidad Autónoma de Nayarit, Compostela 67300, Mexico; 5 Department of Animal and Food Sciences, Texas Tech University, P.O. Box 42141, Lubbock, TX 79409, USA; 6 IPOA Research Group, Centro de Investigación e Innovación Agroalimentaria y Agroambiental, Miguel Hernández University (CIAGRO-UMH), Orihuela, 03312 Alicante, Spain

**Keywords:** edible mushrooms, antioxidant, meat products, shelf life, gastrointestinal digestion

## Abstract

Lipid and protein oxidation are the major causes of meat quality deterioration. Edible mushrooms have been proposed as a strategy to prevent quality deterioration during cold storage. This study aimed to assess the effects of *Pleurotus ostreatus* powder (POP) on the oxidative stability of pork patties during cold storage and after in vitro gastrointestinal digestion (ivGD). Pork patties were subjected to four treatments: control (without antioxidant), T1 (2% POP, *w*/*w*) and T2 (5% POP, *w*/*w*), and T3 as positive control (0.02% BHT, fat basis). POP aqueous, ethanolic, and aqueous ethanol extract were subjected to phytochemical and antioxidant assays. Raw pork patties were subjected to a chemical proximate composition evaluation. At the same time, raw and cooked pork patties were stored at 2 °C for 9 days and subjected to meat quality measurements. Furthermore, the total antioxidant activity of cooked pork patties was determined after ivGD. Results showed that POP ethanol extract showed the highest polysaccharide, phenol, and flavonoid content, as well as antiradical and reducing power properties. POP incorporation into raw and cooked pork patties enhances meat quality traits, including pH, water-holding capacity, cooking-loss weight, texture, color, lipid, and protein oxidation (*p* < 0.05). Furthermore, incorporating POP into cooked samples increases the phytochemical content and antioxidant activity during ivGD. In conclusion, POP has great potential as a natural antioxidant for meat products.

## 1. Introduction

Meat is considered an important source of nutrients for the human diet and food beneficial for consumer health. Meat and meat products contain important levels of proteins, vitamins, minerals, and essential fatty acids, which are indispensable during growth and development, as well as for the maintenance of consumers’ physiological and psychological health [1,2]. Nevertheless, associated with its chemical composition (high proportions of polyunsaturated fatty acids in membrane phospholipids and low presence of endogenous antioxidants) as an intrinsic factor, the formation of free radicals during processing or storage leads to oxidative damage of lipids and proteins. The oxidation of these components is considered the main cause of meat and meat products’ quality loss and may negatively affect nutritional value and physicochemical and sensory properties [3,4].

Thus, synthetic antioxidants, including butylated hydroxyanisole (BHA), butylated hydroxytoluene (BHT), and tert-butylhydroquinone (TBHQ), have been widely used in the meat industry to reduce the lipid and protein oxidation process. However, consumer concerns about usage and safety have been raised by ingesting these additives [5]. In this regard, powders and extracts obtained from plants have been extensively used to extend shelf-life by increasing the oxidative stability of meat and meat products [6] and have contributed to developing healthier and functional meat products [4,6]. In the same way, there has been a trend toward using edible mushrooms as a natural food ingredient to enhance nutritional, sensory, nutraceutical, or functional characteristics [7]. In this context, *Boletus aerus* has been proposed as an ingredient to reduce food-borne pathogens (*Staphylococcus aureus*, *Listeria monocytogenes*, *Escherichia coli,* and *Salmonella typhimurium*) on pork meat during storage at 4 °C for 7 days [8]. Furthermore, *Flammulina velutipes* stem wastes have been considered as a functional ingredient for goat meat nuggets to improve emulsion stability, dietary fiber, ash, and phenolic compounds without affecting textural and sensory parameters (appearance, color, flavor, juiciness, and overall acceptability), as well as antioxidant stability during storage at 4 °C for 9 days [9]. In another work, *Boletus edulis* has been incorporated into beef patties to decrease lipid peroxidation during storage at 4 °C for 8 days [10].

*Pleurotus ostreatus* (oyster mushroom) is a saprophytic edible mushroom species distributed worldwide in temperate and tropical environments and popularly known as the “oyster mushroom” [7,11]. Several investigations evidenced the inclusion of *Pleurotus* spp. in meat products like paste, patties, and sausages, among others, to improve their chemical composition and functional health-promoting properties, as well as to increase physicochemical and sensory properties [12]. For example, it has been demonstrated that *P*. *ostreatus* improves oxidative stability on beef patties during storage at 4 °C for 13 days, which was associated with the phenolic components [13]. Furthermore, it has been demonstrated to possess some phytochemicals like peptides, terpenoids, polysaccharides, and phenolic compounds, which contributed to biological activities such as antihypertensive, anti-inflammatory, antiviral, antinociceptive, immunomodulatory, antimicrobial, and antioxidant [7,11].

Moreover, when natural ingredients are incorporated into meat and meat products, it is important to maintain the presence of phytochemicals during the cooking process and through gastrointestinal digestion after being consumed to provide the functionality to the product and increase the ability to confer beneficial properties to consumer health [14,15]. A previous study revealed that including chia seeds and goji berry puree enhanced the antioxidant capacity of beef patties before and after digestion, which was associated with the presence of polyphenols during the cooking and digestion process [15]. In addition, it has been reported that *Pleurotus eryngii* during simulated gastrointestinal digestion can be used as a functional food to promote intestinal health and prevent disease [16]. However, data on the effect of *P*. *ostreatus* addition on pork patties during cold storage and before and after in vitro gastrointestinal digestion are still limited.

Therefore, the present study was designed to evaluate the effect of *P*. *ostreatus* powder (POP) on the oxidative stability of pork patties during storage and in vitro gastrointestinal digestion (ivGD).

## 2. Materials and Methods

### 2.1. Chemicals and Reagents

All reagents used were of analytical grade. Folin–Ciocalteu reagent, phenol solution, sodium carbonate (Na_2_CO_3_), 1,1-diphenyl-2-picrylhydrazyl (DPPH^•^), 2,2′-Azino-bis(3-ethylbenzothiazoline-6-sulfonic acid) diammonium salt (ATBS), methanol and ethanol (HPLC grade), potassium ferricyanide (K₃[Fe(CN)₆]), potassium persulfate (K_2_S_2_O_8_), potassium hydroxide (KOH), 2,4,6-tri(2-pyridyl)-s-triazine (TPTZ), 2,4-dinitrophenylhydrazine (DNPH), guanidine HCl, iron(III) chloride 6-hydrate (FeCl_3_^•^6H_2_O), iron(II) sulfate 7-hydrate (FeSO_4_^•^7H_2_O), acetic acid (CH_3_COOH), hydrochloric acid (HCl), sulfuric acid (H_2_SO_4_), sodium hydroxide (NaOH), aluminum chloride (AlCl_3_), glucose, gallic acid, tetramethoxypropane (TMP), butylated hydroxytoluene (BHT), and rutin were purchased from Sigma Chemicals (St. Louis, MO, USA). Pancreatin, lipase enzymes, and Oxgall media were obtained from Merck (Darmstadt, Germany). Trichloroacetic (TCA) and 2-thiobarbituric acid (2-TBA) were supplied by JT Baker (Baker ^®^, Phillipsburg, NJ, USA). POP was purchased from Aloha Medicinals, Inc.

### 2.2. Phytochemicals and Antioxidant Activity of POP

#### 2.2.1. Phytochemical Extraction

Phytochemicals from POP were extracted using water, ethanol, and a mixture of water and ethanol–1:1 (1:10, *w*/*v*) as solvent by ultrasound-assisted method (at room temperature, 25 °C, 42 kHz for 1 h). The resultant mixture was filtered (Whatman no. 1 filter paper) under vacuum (vacuum pump MVP 6, Jeju, Republic of Korea), concentrated under reduced pressure at 60 °C for 120 rpm (rotary evaporator Yamato RE301BW, Tokyo, Japan), and lyophilized (freeze dryer Yamato DC401, Tokyo, Japan). The obtained POP extracts were stored at −20 °C in the dark until analysis [17].

#### 2.2.2. Phytochemical Content

The total polysaccharide content (TPLC) of POP was determined by the UV–sulfuric acid method [18]. An aliquot of the extract (0.25 mL, 5 mg/mL) was mixed at 3200 rpm for 1 min (homogenizer Analog Vortex Mixer, Fisher Scientific, Fairlawn, OH, USA) with 0.125 mL of aqueous phenol solution (5%, *v*/*v*) and 0.625 mL of H_2_SO_4_ (concentrated). The reaction mixture was incubated (in the dark, at 25 °C for 20 min), and the absorbance was measured at 490 nm in a spectrophotometer (Multiskan FC UV–Vis, Thermo Scientific, Vantaa, Finland). The results were expressed as mg of glucose equivalents/g of dried extract (mg GE/g).

The total phenolic content (TPC) of POP extracts was determined by the Folin–Ciocalteu method [19]. An aliquot of the extract (5 µL, 5 mg/mL) was homogenized with 80 µL of distilled water, 20 µL of Folin–Ciocalteu reagent (2 M), and 60 µL of Na_2_CO_3_ (5%, *w*/*v*). The reaction mixture was incubated (in the dark, at 25 °C for 1 h), and the absorbance was measured at 730 nm. The results were expressed as mg of gallic acid equivalents/g (mg GAE/g).

The total flavonoid content (TFC) of POP extracts was determined by the aluminum chloride method [20]. An aliquot of the extract (10 µL, 5 mg/mL) was homogenized with 260 µL of methanol and 10 µL of AlCl_3_ (10%, *w*/*v*). The reaction mixture was incubated (in the dark, at 25 °C for 30 min), and the absorbance was measured at 412 nm. The results were expressed as mg of rutin equivalents/g (mg RE/g).

#### 2.2.3. Antioxidant Assays

Free radical scavenging activity (FRSA) of POP extracts was determined by the DPPH^•^ method [21]. An aliquot of the extract (100 µL, 100 µg/mL) was homogenized with 100 µL of DPPH^•^ ethanol solution (300 µM). The reaction mixture was incubated (in the dark, at 25 °C for 30 min), and the absorbance was measured at 517 nm. FRSA was calculated as [(Cabs—Sabs)/Cabs] × 100, where Cabs is the absorbance of the control (t = 0 min), and Sabs is the absorbance of the sample (t = 30 min).

Radical cation scavenging activity (RCSA) of POP extracts was determined by the ABTS^•+^ method [22]. Prior to analysis, equal parts of ABTS ethanol solution (7 mM) and K_2_S_2_O_8_ (2.45 mM) were homogenized and incubated (25 °C, in the dark for 16 h). The obtained mixture was diluted with ethanol until it reached an absorbance of 0.8, and afterwards mixed for 1 min with an aliquot of the extract (ratio 99:1, 5 mg/mL). The reaction mixture was incubated (in the dark, at 25 °C for 6 min), and absorbance was read at 734 nm. RCSA was calculated as [(Cabs—Sabs)/Cabs] × 100, where Cabs is the absorbance of the control (t = 0 min), and Sabs is the absorbance of the sample (t = 6 min).

The reducing power ability (RPA) of POP extracts was determined by Ferricyanide/Prussian blue method [23]. An aliquot of the extract (100 µL, 5 mg/mL) was homogenized with 300 µL of phosphate buffer (0.2 M, pH 6.6) and 300 µL of K₃[Fe(CN)₆] (1%, *w*/*v*). The reaction mixture was incubated in a water bath (in the dark, at 50 °C for 20 min), homogenized with 300 µL of TCA (10%, *w*/*v*), and centrifuged at 4200× *g* at 4 °C for 10 min (Sorvall ST18R, Thermo Fisher Scientific, Waltham, MA, USA). Then, the supernatant was mixed with 100 µL of distilled water and 250 µL of FeCl_3_ (0.1%, *w*/*v*), and the absorbance was measured at 700 nm. The results were expressed as absorbance increased at the same wavelength.

The ferric reducing/antioxidant power assay of POP extracts was determined by the FRAP method [24]. An aliquot of the extract (5 µL, 5 mg/mL) was homogenized with 150 µL of FRAP solution [10:1:1, 300 mM buffer sodium acetate in glacial acetic acid at pH 3.6 and TPTZ (10 mM) in HCl (40 nM) and FeCl_3_ (20 mM)]. The reaction mixture was incubated (in the dark, at 25 °C for 8 min), and the absorbance was measured at 595 nm. The results were expressed as µg of Fe(II) equivalent/g (µg Fe^2+^/g).

### 2.3. Preparation of Pork Patties

Pork meat (*Semimembranosus* m., 48 h post-mortem) was purchased from a local processor (Hermosillo, Mexico), trimmed of all visible extramuscular fat, and minced using a conventional meat grinder (model 4152, Hobart Dayton, OH, USA) that had been equipped with a 4.5 mm orifice plate. Minced meat was mixed (hand mixer model MM25, LEM, OH, USA) with salt (1.5%, *w*/*w*) and fat (10% in the final formulation, *w*/*w*). In each replication (thrice), the mass was divided into four different treatments (36 patties per treatment): Control (without antioxidant); T1, POP at 2% (*w*/*w*); T2, POP at 5% (*w*/*w*); T3, BHT at 0.02% (fat basis). Raw and cooked patties (preheated grill at 180 °C until they reach an internal temperature of 71 °C) were shaped using a manual patty former and placed on a Styrofoam tray. The trays with pork patties were wrapped with polyvinyl chloride film (17,400 cm^3^ O_2_/m^2^ at 23 °C for 24 h). Patties of each treatment were assessed at each sampling point (0, 3, 6, and 9 days). The patties were stored in the dark at 2 °C for 9 days.

### 2.4. Meat Quality Measurements

#### 2.4.1. Physicochemical Assays

The proximate chemical composition of POP and pork patties was determined following the AOAC standard procedures [25], including moisture by oven drying (method 930.30), crude fat content by extraction with petroleum ether in a Goldfish apparatus (method 985.01), total protein content by Kjeldahl apparatus (method 968.06), and ash content by incinerating dried samples at 550 °C. The total carbohydrate content was estimated in percentage as follows: 100% − [moisture content + protein content + crude fat content + ash content].

Pork patties were homogenized with distilled water (1:10, *w*/*v*) at 4500 rpm for 1 min (Ultra-Turrax model T25, IKA^®^, Staufen, Germany) in an ice bath (4 °C), and pH values were measured with a potentiometer with automatic temperature control (Model pH211, Hanna Instruments Inc., Woonsocket, RI, USA) [25].

Before color evaluation, the packaging material was removed, and the samples were exposed to atmospheric O_2_ in the dark, at 0 °C for 30 min, to stabilize the color. After that, ten measurements on sample surfaces were performed using a spectrophotometer (model CM 508d, Konica Minolta Inc., Tokyo, Japan) with a D65 illuminant and 10° observer, which was calibrated with a white calibration cap (CM-A70). Recorded parameters consisted of lightness (L*), redness (a*), yellowness (b*), Chroma (C*), and hue angle (h*) [26].

Pork patties (10 g) were placed on fine mesh nylon, inserted into 50 mL tubes with a screwcap, and centrifuged (4200× *g* at 4 °C for 10 min). The water-holding capacity (WHC) of pork patties was determined gravimetrically [27]. The WHC was expressed as a percentage and calculated as [(initial weight—weight after centrifugation)/initial weight] × 100.

The cooking-loss weight (CLW) of pork patties was determined after cooking the samples in a preheated grill at 180 °C (until they reach an internal temperature of 70 °C) [28]. The CLW was expressed as a percentage and calculated as [(weight before cooking—weight after cooking)/weight before cooking] × 100.

Fracture texture profile analysis of pork patties was measured in a Texture Analyzer (model TAXT Plus kit, Stable Micro System, Ltd. Godalming, Surrey, UK). Before texture analysis, pork patties (120 g each, 10 cm diameter × 1 cm height) were exposed to atmospheric O_2_ at 25 °C until they reached an internal temperature of 25 °C. The setting was distance = 40 mm, pre-test speed = 60 mm/min, post-test speed = 600 mm/min, head speed = 100 mm/min, and force = 5 g. The fracturability values were expressed as kg force (kg–f) [29].

#### 2.4.2. Oxidative Stability

Lipid oxidation was measured by the thiobarbituric acid reactive substances (TBARS) method [30]. Pork patties (10 g) were homogenized in an ice bath (4 °C) with 20 mL TCA (10%, *w*/*v*) at 4500 rpm for 1 min. Afterwards, the slurry was centrifuged (2300× *g* at 4 °C for 20 min) and filtered (Whatman 4 filter paper). In total, 2 mL of the filtered supernatant was mixed with 2 mL of 2-TBA (0.02 M), placed in a water bath at 98 °C for 20 min, subsequently cooled at 25 °C, and the absorbance was measured at 531 nm against a water blank. TBARS values were calculated from a 1, 1, 3, 3-tetramethoxypropane standard curve. The results were expressed as mg malondialdehyde/kg of the meat sample (mg MDA/kg).

The protein oxidation was measured by the total carbonyl content method [31]. Pork patties (1 g) were homogenized (1:10, *w*/*v*) in sodium phosphate buffer (20 mM) containing NaCl (0.6 M, pH 6.5) at 4500 rpm for 1 min. Two equal aliquots of 0.2 mL were taken from homogenates, and each was dispensed in 2 mL test tubes. Proteins were precipitated with 1 mL of cold TCA (10%, *w*/*v*) and subsequently centrifuged at 4200× *g* at 4 °C for 5 min [one pellet was treated with 1 mL of HCl (2 M) for protein concentration measurement, and the other with an equal volume of DNPH in HCl (2 M) for carbonyl concentration measurement]. Both samples were incubated at 25 °C for 1 h. Afterwards, samples were precipitated with 1 mL of TCA (10%, *w*/*v*) and washed twice with 1 mL of ethanol:ethyl acetate (1:1, *v*/*v*) to remove excess DNPH. Then, each pellet was mixed with 1.5 mL of sodium phosphate buffer (20 mM) containing guanidine HCl (6 M, pH 6.5), stirred, and centrifuged at 4200× *g* at 4 °C for 2 min to remove insoluble fragments. Protein concentration was calculated from absorption at 280 nm using BSA as the standard. The number of carbonyls was expressed as nM of carbonyl per mg of protein using a molar absorption coefficient of 21 nM^−1^ × cm^−1^ at 370 nm for protein hydrazones.

### 2.5. ivGD of Pork Patties

The ivGD of cooked pork patties was simulated in the presence or not of a basal diet (20% of lipids, 15% of protein, and 45% of carbohydrates) for a healthy adult (ivGD1 and ivGD2, respectively) [32]. The physiological solution was prepared with distilled water (200 mL) and placed in a screw-top flask inside a shaking incubator (Inforst HT, Ecotron, Switzerland) at 150 rpm until it reached an internal temperature of 37 °C. Afterwards, pH was adjusted between 2.0–2.5 with HCl (5 M), while the stomach phase was simulated by adding 0.33 g of pepsin at a 1:10,000 (enzyme-substrate) ratio. Subsequently, minced pork patties (120 g) were mixed with 200 mL of physiological solution and subjected to a continuous digestion process (150 rpm at 37 °C for 2 h). Then, 50 mL of the resultant mixture was adjusted to a pH of 5.0–5.5 with NaOH (3 M) to inactivate the enzyme. At the same time, the small intestine phase was simulated by adding 50 mL of distilled water containing 0.19 g of pancreatin (25,000 UI), 0.001 g of lipase (type II, 100–500 units/mg of protein), and 1 g of Oxgall. After digestion (150 rpm at 37 °C for 4 h), enzyme activity was inhibited by heating at 95 °C for 10 min. The digested sample was centrifuged (4200× *g* at 4 °C for 20 min) and filtered (Millipore filter 0.2–0.4 µm). Then, the resulting supernatant was employed to carry out the phytochemical and antioxidant activity measurements.

### 2.6. Statistical Analysis

Three independent experimental trials were conducted, and results were expressed as mean ± standard deviation (SD). Normal distribution and variance homogeneity were previously tested (Shapiro–Wilk). Phytochemical and antioxidant data obtained were subjected to one-way analysis of variance (ANOVA) with the fixed effect for extraction solvent (water, ethanol, and a mixture of water and ethanol in a ratio 1:1). Meat quality data were submitted to two-way ANOVA, with the antioxidant treatment (control, T1, T2, and T3) and storage time (0, 3, 6, and 9 days) as the main effects and the two-way interaction, while ivGD data considered the treatments (control, T1, T2, and T3) and the phase (gastric and intestinal) as the fixed terms in the model. A Tukey–Kramer multiple comparison test was performed to determine the significance of mean values for multiple comparisons at *p* < 0.05. All data were analyzed using statistical software (SPPS version 21).

## 3. Results and Discussion

### 3.1. Phytochemical Content and Antioxidant Activity of POP

The phytochemicals such as alkaloids, steroids, terpenes, glycosides, phenolic acids, and flavonoids are widely distributed in nature and can be obtained by consuming fruits, vegetables, seeds, and edible mushrooms, among other sources [6,7,11]. These compounds have the potential to protect different food matrices against damage caused by spoilage bacteria and by the oxidation reactions implicit in the product [6]. However, it has been demonstrated that the extraction method combined with several factors (pressure, temperature, time, solute–solvent ratio, solvent polarity, among others) can affect the type and concentration of the obtained phytochemical [33,34,35].

Respecting phytochemical content, as shown in Table 1, the results indicated that the highest (*p* < 0.05) TPLC value was showed by POP ethanol extract (approx. 15 mg GE/g), and POP ethanol and aqueous ethanol (1:1) extracts presented the highest TPC (approx. 40 mg GAE/g), while POP ethanol extract showed the highest TFC (approx. 20 mg RE/g). In addition, the results of the antioxidant activity also showed that the highest (*p* < 0.05) RCSA, RPA, and FRAP values were observed by the used standard (BHT), although when comparing between extracts, POP extracts obtained with ethanol showed higher (*p* < 0.05) RCSA, RPA, and FRAP values (>50% of inhibition, >0.4 abs and >0.6 µg Fe^2+^/g, respectively). Furthermore, POP aqueous, ethanol, and 1:1 extract showed higher FRSA (>90% of inhibition) than BHT (*p* < 0.05).

In agreement with our work, a higher phenolic content was observed in *P*. *ostreatus* extracts when ethanol and a mixture of water-ethanol were used as solvent extraction, in comparison with aqueous extracts [36]. In contrast with our work, a higher polysaccharide and phenolic compound extraction have been reported when water was used as solvent extraction than a reduced polar solvent [33,34]. Concerning antioxidant results, in agreement with our study, no significant differences in FRSA values were showed in *P*. *ostreatus* extracts obtained with different solvents, including water, ethanol, and a mixture of water-ethanol [36].

In contrast, no significant differences were observed in RCSA and RPA of *P*. *ostreatus* extracts recovered with different solvents [36]. Furthermore, in a previous report [33], the highest FRAP values were obtained in *P*. *ostreatus* aqueous extracts compared to methanol extracts. Moreover, no differences were observed in RCSA values by the effect of solvent extraction; however, in agreement with our study, the highest FRSA and RPA values were found in methanol extracts.

### 3.2. Physicochemical Analysis of Meat Samples

Moreover, incorporating natural powers and extracts into raw and cooked meat products has been considered an important strategy to modify the chemical composition, increase oxidative stability during storage, and improve meat quality parameters [37,38]. As shown in Table 2, the results showed that the major components of dried POP were mainly carbohydrates, followed by proteins and fats, and in trace amounts, ash and moisture (*p* < 0.05). Regarding the chemical composition of meat samples, no significant differences (*p* > 0.05) were shown in the fat protein and ash content of pork patties. On the other hand, T2 samples showed the lowest moisture content values, and T1 and T2 had the highest carbohydrate content values (*p* < 0.05). In agreement with our work, it has been reported that the inclusion of POP (5%) in raw beef patties significantly improves carbohydrate content [13], which could be associated with the fact that these are major components found in the powder [39,40].

As shown in Table 3, at the initial day of storage (day 0), no significant differences (*p* > 0.05) were shown in pH values between treatments of raw and cooked pork patties. These values significantly decreased during storage time (*p* < 0.05). At the end of storage (day 9), raw pork patties treated with T1, T2, and T3 showed higher (*p* < 0.05) pH values than control samples, while in cooked samples, no significant differences (*p* > 0.05) were shown in pH values between treatments (*p* < 0.05). Moreover, no significant differences (*p* > 0.05) were shown in the initial WHC values of raw pork patties between treatments, while T2 showed the highest WHC in cooked samples, concerning control samples (*p* < 0.05). These values significantly decreased during storage time (*p* < 0.05), and on day 9, T1 and T2 showed the highest WHC values of raw and cooked pork patties. Respecting CLW, at days 0 and 9, T1 and T2 showed the lowest (*p* < 0.05) values in comparison to the other treatments. Additionally, at day 0, no differences were found in texture values between treatments of raw and cooked pork patties, while on day 9, T1 and T2 showed the lowest (*p* < 0.05) texture values.

Although a reduction in initial pH values was observed in raw pork patties by the effect of 2 and 5% of POP incorporation (T1 and T2, respectively), pH values remained (5.6–5.9) within the characteristic value of fresh meat [28]. Similarly, a reduction in initial pH values (1%) was observed in raw beef patties incorporated with 4% of edible mushroom powder [39]. In contrast, the initial pH value (5.3) was not modified in raw beef patties treated with 3 and 5% of edible mushroom extracts (*Boletus edulis*) [10]. On the other hand, an increase in initial pH values of cooked pork patties was observed among treatments, which is associated with the decomposition of the cellular buffer system and the release of fat during cooking [41]. Our results indicate that pH values of raw patties treated with T1 and T2 were stable during the storage period, compared to control and T3 samples. These findings agree with those previously observed by other investigations [13].

In this regard, the pH is a major parameter related to meat and meat products’ quality due to its significant impact on physicochemical (WHC, CLW, texture, and color) and oxidative stability (lipid and protein oxidation) [13,28,42]. The WHC is a quality parameter of meat and meat products, indicating their capacity to retain water when exposed to external forces such as cutting, grinding, pressing, and heating, while CLW indicates the weight yield of meat or meat products after cooking. Furthermore, it has been reported that a decreased WHC and increased CLW values are associated with a greater hardness (texture) of the meat and meat products [28,43]. Our study also demonstrated that WHC and texture values of raw and cooked pork patties treated with T1 and T2 were stable during storage compared to control samples, as well as the CLW of cooked samples. These findings agree with those previously reported [13], which indicate that the inclusion of POP (4%) in raw beef patties decreased CLW and texture (13.0 and 17.5%, respectively) during storage at 4 °C for 13 days. In addition, an increase in water retention has been demonstrated in emulsion-type sausages treated with winter mushroom powder [43].

The color of meat and meat products is an important parameter to be considered in the quality evaluation due extensively to the freshness of a product and related to the purchase intention [6,42]. Lightness (L* value) indicates the degree of luminosity or brightness of a color and takes values between 0 to 100 (black to white). While a* and b* (redness and yellowness, respectively) decrease during oxidation, changes are more displayed in a* values. In addition, C* and h* (Chroma and hue angle, respectively) are highly correlated with human visual color perception, but C* values are decreased during oxidation, and h* values are increased [42].

As shown in Table 4, at day 0, raw pork patties treated with T2 showed lower (*p* < 0.05) L* values than another treatment, and no significant differences (*p* > 0.05) were shown between treatments in cooked samples. These values significantly decreased during storage time (*p* < 0.05). On day 9, T2 showed the lowest (*p* < 0.05) values for raw samples and T1 and T2 for cooked samples, concerning control samples. Moreover, at day 0 and day 9, T1 and T2 showed the highest (*p* < 0.05) a* values in raw and cooked patties, in comparison with other treatments. Respecting b* values, T1 and T2 presented the highest (*p* < 0.05) values during all storage periods in raw samples; however, on day 9, no differences (*p* > 0.05) were found in this parameter between treatments in cooked samples. Furthermore, on day 9, T1 and T2 showed the highest (*p* < 0.05) C* values in raw patties, without differences during storage for cooked samples (*p* > 0.05). In addition, at day 0, no differences (*p* > 0.05) were found in h* values between treatments in raw and cooked samples. On day 9, T1 and T2 showed the lowest h* values in raw and cooked samples. In agreement with a previous report [13], the incorporation of 5% of *P*. *ostreatus* powder increased a* and b* values of raw beef patties at 4 °C for 13 days. In contrast, in the same study, no significant differences were shown in the L* values of samples treated with the mushroom.

### 3.3. Oxidative Stability of Meat Samples

**Figure 1:**
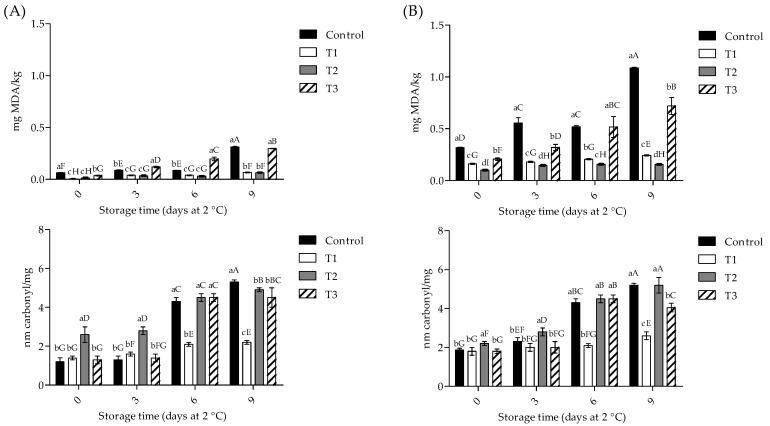
Lipid and protein oxidation of raw and cooked pork patties ((**A**) and (**B**), respectively) during storage time. T1, POP at 2%; T2, POP at 5%; T3, BHT at 0.02%. Lowercase letters indicate differences between treatments on each sampling day; capital letters indicate differences in each treatment through the storage period (*p* < 0.05).

Meat and meat products are extensively susceptible to lipid and protein oxidation processes and, consequently, to quality deterioration during storage. In contrast, to delay or inhibit both processes, natural extracts and powders have been proposed as food ingredients [6,30,31]. As shown in Figure 1, at day 0, raw and cooked pork patties treated with T1 and T2 showed lower (*p* < 0.05) TBARS values than control and T3 samples. On day 9, T1 and T2 showed the lowest (*p* < 0.05) TBARS values for raw samples and T2 for cooked samples, concerning control samples. Regarding protein oxidation, T2 presented the highest carbonyl content values (*p* < 0.05) for raw and cooked patties, in comparison with other treatments. On day 9, T2 showed the lowest carbonyl content values for raw samples, while T1 in cooked samples, in comparison to control samples. These oxidation values significantly increased during storage time (*p* < 0.05).

In agreement with our study, a reduction in initial MDA values (83.8% by both) was reported for raw beef patties treated with 3 and 5% of edible mushroom extracts (*Boletus edulis*) [10]. Furthermore, a lower MDA and carbonyl content was also reported in beef salami treated with 1%, 2%, and 3% of *P*. *ostreatus* during storage than control samples, in concentration dependence [44]. In contrast, a lower MDA content was reported in an emulsion-type sausage treated with 1% and 2% of *P*. *ostreatus* during storage [45].

In contrast, it has been reported that raw beef patties incorporated with 5% of *P*. *ostreatus* powder increased MDA formation (9.5%) during storage at 4 °C for 13 days; however, in the same study, it was observed to decrease in MDA formation (30.1%) on samples treated with the mushroom at day 6 of storage [13]. Furthermore, it has been reported that there are no differences in the carbonyl content of meat samples by thermal effect between treatments [46].

Although data on the effect of edible mushroom addition on meat products against lipid oxidation are still limited, it has been demonstrated that there is a positive effect of natural sources against these parameters on meat products [6].

### 3.4. Phytochemical Content and Antioxidant Activity of Meat Samples Subjected to ivGD

ivGD models are widely used to study the structural changes, digestibility, and release of meat and meat products’ components before and after digestion [37,47]. In this model, it has been demonstrated that meat from different species delivers important nutrients to humans and provides a source of antioxidant peptides through ivGD. Furthermore, it revealed that muscle type (pork and turkey > beef and chicken) and cooking temperature may affect, quantitatively and qualitatively, the release of bioactive peptides and related bioactivity of digested meat [14].

**Figure 2:**
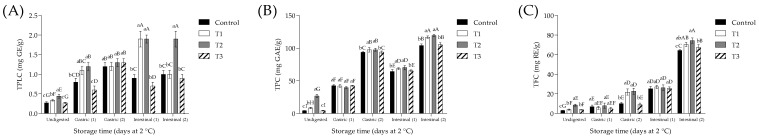
Total polysaccharide, phenolic, and flavonoid contents ((**A**), (**B**), and (**C**), respectively) of cooked pork patties before and after ivGD. T1, POP at 2%; T2, POP at 5%; T3, BHT at 0.02%. Lowercase letters indicate differences between treatments on each sampling day; capital letters indicate differences in each treatment through the storage period (*p* < 0.05).

As shown in Figure 2, the results showed that TPLC, TPC, and TFC were increased (*p* < 0.05) during ivGD (intestinal phase > gastric phase) in comparison to undigested cooked pork patties, mainly when a basal diet was used during ivGD. At the initial step of ivGD1 (gastric phase), T1 and T2 showed the highest TPLC value concerning control and T3, without differences between treatments for TPC and TFC values (*p* > 0.05). Furthermore, at the initial step of ivGD2 (gastric phase), T1 and T2 showed the highest TPC and TFC values in comparison to another treatment, without differences (*p* > 0.05) in TPLC values. At the final step of ivGD1 (intestinal phase), T1 and T2 showed the highest (*p* < 0.05) TPLC and TPC to control and T3, without differences (*p* > 0.05) in TFC. In addition, at the final step of ivGD2 (intestinal phase), T1 and T2 showed the highest TPC and TFC concerning control and T3, while T2 showed the highest TPLC value (*p* < 0.05).

**Figure 3:**
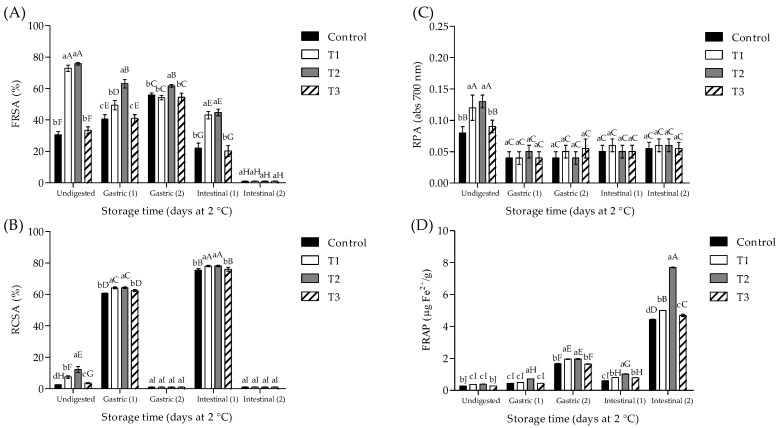
FRSA, RCSA, RPA, and FRAP values ((**A**–**D**), respectively) of cooked pork patties before and after ivGD. T1, POP at 2%; T2, POP at 5%; T3, BHT at 0.02%. Lowercase letters indicate differences between treatments on each digestion step; capital letters indicate differences in each treatment through ivGD (*p* < 0.05).

Moreover, as shown in Figure 3, at the initial step of ivGD1 (gastric phase), T1 and T2 showed the highest (*p* < 0.05) FRSA, RCSA, and FRAP values concerning control and T3, while no significant differences (*p* > 0.05) were observed in RPA between treatments. Furthermore, at the initial step of ivGD2 (gastric phase), T1 and T2 showed the highest (*p* < 0.05) FRSA and FRAP values in comparison to control and T3, without differences (*p* > 0.05) in RCSA and RPA values. At the end step of ivGD1 (intestinal phase), T1 and T2 showed the highest (*p* < 0.05) FRSA, RCSA, and FRAP values in comparison to control and T3, without differences (*p* > 0.05) in RPA values, while at the end step of ivGD2 (intestinal phase), T2 showed the highest (*p* < 0.05) FRAP values in comparison to other treatments, without differences (*p* > 0.05) in FRSA, RCSA, and RPA values.

In the current study, incorporating *P. ostreatus* powder into cooked pork patties improves phytochemical compounds and antioxidant activity during ivGD. In agreement with this, an increase in polyphenol content and antiradical activity (FRSA and RCSA) was shown in cooked beef patties with 2.5 and 5% of chia seeds and goji puree after being subjected to ivGD [15]. Another report [48] evaluated the antioxidant effect of traditional balsamic vinegar on roasted turkey meat subjected to ivGD and reported that phenolic acids and flavonoids could exert metal-chelating activity through the digestion process.

## 4. Conclusions

In this investigation, the current findings demonstrated that *P*. *ostreatus* extracts, mainly ethanolic, are an important source of phytochemicals like total polysaccharides, phenolic, and flavonoids. Regarding antioxidant activities (RCSA, RPA, and FRAP), ethanol extracts exert the highest values without differences in FRSA activity between extracts (water, ethanol, and water-ethanol). About physicochemical properties, the results indicate that incorporation of 2 and 5% *P*. *ostreatus* powder into raw and cooked pork patties improves pH, WHC, CLW, texture, and color stability during storage, in comparison with the control (samples without antioxidants) and BHT (synthetic antioxidant). In addition, raw pork patties treated with 2 and 5% of *P*. *ostreatus* powder showed the lowest lipid and protein oxidation levels compared to the control, while in cooked samples, the lowest lipid and protein oxidation values were presented in samples treated with 5 and 2% of *P*. *ostreatus* incorporation, respectively. Furthermore, incorporating *P. ostreatus* powder into cooked samples increases the phytochemical content and antioxidant activity during ivGD, without showing the effect of the mushroom addition level, except for FRAP values that were higher at the highest addition level.

## Figures and Tables

**Table 1:** Phytochemical content and antioxidant activity of POP extracts.

Item	Test	Water	Ethanol	1:1	BHT
Phytochemical content	TPLC (mg GE/g)	13.0 ± 1.0 ^b^	15.0 ± 1.0 ^a^	10.0 ± 1.0 ^c^	
	TPC (mg GAE/g)	35.1 ± 0.6 ^c^	39.0 ± 1.0 ^b^	40.1 ± 0.6 ^b^	
	TFC (mg RE/g)	18.0 ± 1.2 ^b^	20.1 ± 1.0 ^a^	15.8 ± 0.3 ^c^	
Antioxidant activity	FRSA (%)	91.7 ± 1.7 ^a^	92.0 ± 1.5 ^a^	90.9 ± 0.7 ^a^	70.9 ± 1.3 ^b^
	RCSA (%)	55.7 ± 0.9 ^c^	58.5 ± 1.0 ^b^	56.4 ± 0.5 ^c^	65.8 ± 0.7 ^a^
	RPA (abs)	0.40 ± 0.1 ^a^	0.45 ± 0.1 ^b^	0.41 ± 0.2 ^a^	0.80 ± 0.1 ^c^
	FRAP (µg Fe^2+^/g)	0.65 ± 0.1 ^c^	0.68 ± 0.1 ^b^	0.61 ± 0.1 ^d^	0.74 ± 0.1 ^a^

Values expressed as mean ± SD of at least three independent experiments. POP, *P*. *ostreatus* powder; TPLC, total polysaccharide content; TPC, total phenolic content; TFC, total flavonoids content; FRSA, free-radical scavenging activity; RCSA, radical cation scavenging activity; RPA, reducing power ability; FRAP, ferric reducing/antioxidant power assay; BHT, butylated hydroxytoluene (50 µg/mL). Different superscripts (a–d) in the same row differ significantly (*p* < 0.05).

**Table 2:** Chemical proximate composition of POP and pork patties.

Treatment	Moisture	Fat	Protein	Ash	Carbohydrate
POP	0.9 ± 0.6	9.2 ± 0.9	8.4 ± 0.1	5.2 ± 0.1	76.0 ± 0.9
Control	70.3 ± 1.3 ^a^	9.6 ± 0.8	18.0 ± 1.2	1.8 ± 0.3	0.7 ± 0.2 ^b^
T1	68.8 ± 0.1 ^b^	9.5 ± 0.7	19.2 ± 1.3	1.8 ± 0.3	1.2 ± 0.3 ^a^
T2	65.4 ± 0.4 ^c^	10.0 ± 1.2	20.1 ± 1.6	2.2 ± 0.5	1.3 ± 0.3 ^a^
T3	68.4 ± 1.2 ^ab^	10.1 ± 0.2	19.0 ± 1.3	2.0 ± 0.1	0.9 ± 0.2 ^b^

Values expressed as mean ± SD of at least three independent experiments. POP, *P*. *ostreatus* powder. T1, POP at 2%; T2, POP at 5%; T3, BHT at 0.02%. Different superscripts (a–b) in the same column differ significantly (*p* < 0.05).

**Table 3:** Meat quality attributes of raw and cooked pork patties during storage time.

Item	Meat	Day	Treatments			
			Control	T1	T2	T3
pH	Raw	0	5.8 ± 0.1 ^aA^	5.8 ± 0.2 ^aA^	5.7 ± 0.1 ^aA^	5.9 ± 0.1 ^aA^
		3	5.8 ± 0.1 ^aA^	5.7 ± 0.1 ^aA^	5.7 ± 0.1 ^aA^	5.6 ± 0.1 ^aB^
		6	5.7 ± 0.1 ^aAB^	5.7 ± 0.1 ^aA^	5.7 ± 0.1 ^aA^	5.6 ± 0.1 ^aB^
		9	5.4 ± 0.1 ^bB^	5.7 ± 0.1 ^bA^	5.7 ± 0.1 ^bA^	5.6 ± 0.1 ^abB^
	Cooked	0	6.1 ± 0.1 ^aA^	6.1 ± 0.1 ^aA^	6.1 ± 0.1 ^aA^	6.0 ± 0.1 ^aA^
		3	6.1 ± 0.1 ^aA^	5.9 ± 0.1 ^aAB^	5.9 ± 0.1 ^aA^	5.9 ± 0.1 ^aA^
		6	6.1 ± 0.1 ^aA^	5.9 ± 0.1 ^aAB^	5.9 ± 0.1 ^aA^	5.9 ± 0.1 ^aA^
		9	5.8 ± 0.1 ^aB^	5.8 ± 0.1 ^aB^	5.9 ± 0.1 ^aA^	5.9 ± 0.1 ^aA^
WHC (%)	Raw	0	90.1 ± 2.2 ^aA^	90.4 ± 2.0 ^aA^	92.7 ± 0.5 ^aA^	89.7 ± 0.4 ^aA^
		3	90.0 ± 1.3 ^aA^	91.9 ± 0.8 ^aA^	92.3 ± 1.1 ^aA^	89.0 ± 1.5 ^aA^
		6	91.0 ± 2.0 ^aA^	90.8 ± 1.5 ^aA^	92.2 ± 0.7 ^aA^	85.3 ± 1.3 ^bB^
		9	85.7 ± 1.1 ^bB^	91.9 ± 1.4 ^aA^	92.9 ± 0.4 ^aA^	84.1 ± 0.3 ^bB^
	Cooked	0	85.7 ± 1.9 ^bA^	89.2 ± 1.9 ^bA^	93.9 ± 1.4 ^aA^	89.4 ± 1.4 ^bA^
		3	85.7 ± 1.9 ^bA^	88.1 ± 1.5 ^bA^	94.3 ± 1.8 ^aA^	87.5 ± 1.5 ^bAB^
		6	85.7 ± 1.6 ^aA^	86.1 ± 1.6 ^aA^	84.5 ± 2.0 ^aB^	85.1 ± 1.7 ^aB^
		9	79.2 ± 2.3 ^bB^	86.3 ± 1.4 ^aA^	85.2 ± 2.3 ^aB^	82.1 ± 1.8 ^aB^
CLW (%)	Raw	0	--	--	--	--
		3	--	--	--	--
		6	--	--	--	--
		9	--	--	--	--
	Cooked	0	16.0 ± 1.2 ^aB^	14.9 ± 1.4 ^abB^	11.9 ± 1.9 ^bB^	16.0 ± 0.7 ^aB^
		3	22.5 ± 1.2 ^aA^	16.6 ± 1.3 ^bB^	14.0 ± 1.6 ^bB^	21.9 ± 1.9 ^aA^
		6	21.6 ± 1.5 ^aA^	19.3 ± 0.7 ^bA^	16.3 ± 0.4 ^cA^	22.8 ± 1.3 ^aA^
		9	23.8 ± 2.3 ^aA^	19.4 ± 0.7 ^bA^	17.1 ± 0.7 ^bA^	25.2 ± 1.7 ^aA^
Texture (kg–f)	Raw	0	1.1 ± 0.3 ^aB^	1.0 ± 0.3 ^aA^	1.1 ± 0.3 ^aA^	1.2 ± 0.2 ^aB^
		3	1.1 ± 0.1 ^aB^	1.1 ± 0.2 ^aA^	1.1 ± 0.3 ^aA^	1.1 ± 0.3 ^aB^
		6	1.0 ± 0.2 ^aB^	1.1 ± 0.1 ^aA^	1.2 ± 0.2 ^aA^	1.2 ± 0.2 ^aB^
		9	1.6 ± 0.1 ^aA^	1.2 ± 0.2 ^bA^	1.2 ± 0.2 ^bA^	1.7 ± 0.2 ^aA^
	Cooked	0	0.9 ± 0.1 ^aB^	1.1 ± 0.1 ^aA^	1.0 ± 0.2 ^aA^	0.9 ± 0.1 ^aB^
		3	0.9 ± 0.1 ^aB^	1.0 ± 0.2 ^aA^	1.1 ± 0.2 ^aA^	1.2 ± 0.1 ^abA^
		6	1.0 ± 0.1 ^aB^	1.0 ± 0.2 ^aA^	1.1 ± 0.2 ^aA^	1.2 ± 0.1 ^aA^
		9	1.4 ± 0.1 ^bA^	1.0 ± 0.2 ^aA^	1.1 ± 0.2 ^aA^	1.4 ± 0.1 ^bA^

Values expressed as mean ± SD of at least three independent experiments. POP, *P*. *ostreatus* powder. T1, POP at 2%; T2, POP at 5%; T3, BHT at 0.02%; WHC, water-holding capacity; CLW, cooking-loss weight. Lowercase letters indicate differences between treatments on each sampling day; capital letters indicate differences between each treatment through the storage period (*p* < 0.05).

**Table 4:** Instrumental color during storage of raw and cooked pork patties.

Item	Meat	Day	Treatments			
			Control	T1	T2	T3
L*	Raw	0	49.3 ± 2.3 ^aA^	48.6 ± 1.5 ^aA^	44.7 ± 2.1 ^bA^	48.0 ± 2.5 ^aA^
		3	49.6 ± 2.5 ^aA^	48.1 ± 2.5 ^aA^	45.4 ± 1.2 ^bA^	51.4 ± 1.9 ^aA^
		6	52.4 ± 2.5 ^aAB^	50.0 ± 2.3 ^aA^	45.0 ± 1.1 ^bA^	51.3 ± 2.1 ^aA^
		9	55.0 ± 1.5 ^aA^	50.1 ± 1.4 ^bA^	45.9 ± 2.2 ^cA^	52.1 ± 1.8 ^bA^
	Cooked	0	52.4 ± 2.0 ^aB^	47.9 ± 2.5 ^aA^	49.3 ± 2.5 ^aA^	52.6 ± 2.8 ^aB^
		3	59.4 ± 2.3 ^aA^	51.6 ± 2.5 ^bcA^	48.9 ± 2.5 ^cA^	54.4 ± 2.2 ^bB^
		6	59.3 ± 2.5 ^aA^	50.5 ± 3.0 ^bcA^	49.0 ± 2.7 ^cA^	55.3 ± 2.5 ^bAB^
		9	60.3 ± 1.6 ^aA^	53.7 ± 2.7 ^bA^	53.0 ± 2.8 ^bA^	59.0 ± 2.7 ^aA^
a*	Raw	0	7.9 ± 0.9 ^bA^	10.1 ± 1.5 ^aA^	11.6 ± 1.0 ^aA^	7.3 ± 1.1 ^bA^
		3	6.8 ± 1.2 ^bAB^	10.0 ± 1.5 ^aA^	10.6 ± 0.8 ^aA^	6.5 ± 0.8 ^bAB^
		6	5.1 ± 1.1 ^bBC^	8.9 ± 1.5 ^aA^	10.0 ± 0.9 ^aA^	6.4 ± 0.7 ^bAB^
		9	4.8 ± 0.6 ^bC^	8.3 ± 1.0 ^aA^	9.9 ± 0.9 ^aA^	5.1 ± 1.0 ^bB^
	Cooked	0	4.9 ± 1.1 ^bA^	8.2 ± 1.3 ^aA^	8.2 ± 1.4 ^aA^	7.6 ± 1.5 ^abA^
		3	4.1 ± 1.3 ^bA^	7.8 ± 1.4 ^aA^	8.0 ± 1.1 ^aA^	6.0 ± 1.3 ^aAB^
		6	3.5 ± 0.7 ^bA^	7.0 ± 1.0 ^aA^	7.2 ± 1.0 ^aA^	6.1 ± 0.8 ^aAB^
		9	2.7 ± 0.8 ^bA^	6.1 ± 1.2 ^aA^	6.5 ± 1.2 ^aA^	4.4 ± 1.1 ^abB^
b*	Raw	0	14.2 ± 1.3 ^bA^	19.2 ± 1.1 ^aA^	21.9 ± 1.5 ^aA^	14.1 ± 1.2 ^bA^
		3	13.6 ± 1.5 ^bA^	18.9 ± 1.4 ^aA^	20.5 ± 1.1 ^aA^	12.6 ± 0.6 ^bA^
		6	13.0 ± 1.5 ^bA^	19.0 ± 1.5 ^aA^	20.6 ± 1.1 ^aA^	13.6 ± 0.6 ^bA^
		9	12.4 ± 0.5 ^bA^	18.9 ± 1.6 ^aA^	20.1 ± 1.6 ^aA^	13.1 ± 1.5 ^bA^
	Cooked	0	19.2 ± 2.3	21.2 ± 2.4	22.4 ± 1.8	22.2 ± 1.4
		3	20.0 ± 1.3	23.4 ± 1.2	21.9 ± 1.3	22.0 ± 2.4
		6	19.6 ± 1.9	22.0 ± 1.9	20.9 ± 1.2	20.5 ± 1.1
		9	20.8 ± 1.7	21.5 ± 1.8	20.7 ± 1.4	22.2 ± 1.0
C*	Raw	0	15.9 ± 1.2 ^bA^	21.5 ± 1.4 ^aA^	24.0 ± 1.9 ^aA^	15.8 ± 1.4 ^bA^
		3	15.2 ± 1.5 ^bA^	21.4 ± 2.5 ^aA^	23.1 ± 1.2 ^aA^	15.2 ± 0.6 ^bA^
		6	14.0 ± 1.5 ^bA^	21.0 ± 2.2 ^aA^	22.9 ± 1.3 ^aA^	14.4 ± 1.4 ^bA^
		9	12.2 ± 0.5 ^bB^	21.0 ± 1.4 ^aA^	22.3 ± 1.8 ^aA^	14.3 ± 1.4 ^bA^
	Cooked	0	20.4 ± 1.4 ^bA^	25.0 ± 1.4 ^aA^	24.0 ± 0.9 ^aA^	24.9 ± 1.0 ^aA^
		3	20.5 ± 1.5 ^bA^	24.7 ± 1.4 ^aA^	23.4 ± 1.4 ^aA^	24.1 ± 1.1 ^aA^
		6	19.0 ± 1.5 ^bA^	23.1 ± 1.0 ^aAB^	21.8 ± 1.4 ^aA^	23.7 ± 1.1 ^aAB^
		9	19.0 ± 1.6 ^aA^	21.8 ± 1.0 ^aB^	21.8 ± 1.3 ^aA^	21.0 ± 1.3 ^aB^
h*	Raw	0	64.0 ± 2.0 ^aB^	62.8 ± 1.9 ^aA^	61.1 ± 1.4 ^aB^	62.8 ± 2.2 ^aA^
		3	63.4 ± 2.4 ^aB^	62.2 ± 2.4 ^aA^	62.6 ± 1.5 ^aAB^	63.5 ± 2.3 ^aA^
		6	70.4 ± 2.5 ^aA^	65.1 ± 2.2 ^bA^	63.0 ± 1.5 ^bAB^	64.6 ± 2.5 ^bA^
		9	70.0 ± 1.3 ^aA^	66.4 ± 1.8 ^bcA^	63.0 ± 1.1 ^cA^	67.6 ± 2.0 ^ab^
	Cooked	0	73.2 ± 2.5 ^aB^	71.8 ± 2.5 ^aA^	71.4 ± 2.4 ^aA^	72.1 ± 2.3 ^aB^
		3	78.6 ± 2.3 ^aA^	71.5 ± 2.5 ^bA^	70.1 ± 2.4 ^bA^	74.9 ± 2.1 ^abAB^
		6	81.4 ± 2.5 ^aA^	72.3 ± 2.5 ^bA^	73.2 ± 2.4 ^bA^	75.8 ± 1.4 ^bAB^
		9	80.6 ± 1.3 ^aA^	74.3 ± 2.6 ^bA^	72.0 ± 1.0 ^bA^	77.1 ± 2.6 ^aA^

Values expressed as mean ± SD of at least three independent experiments. POP, *P*. *ostreatus* powder. T1, POP at 2%; T2, POP at 5%; T3, BHT at 0.02%; WHC, water-holding capacity; CLW, cooking-loss weight. Lowercase letters indicate differences between treatments on each sampling day; capital letters indicate differences between each treatment through the storage period (*p* < 0.05).

## Data Availability

Data is contained within the article.
